# Supplementary material for: Magnesium Depletion Score as an Indicator of Health Risk and Nutritional Status—A Scoping Review
Source: Nutrients. 2025 Oct 20;17(20):3286. doi: 10.3390/nu17203286 (PMC12566843; doi:10.3390/nu17203286)
Supplement: Supplementary file 1 [file nutrients-17-03286-s001.zip › nutrients-3891885-supplementary (final).pdf]

Supplemental Material

Magnesium Depletion Score as an Indicator of Health Risk and Nutritional Status—A Scoping Review

Table S1. Literature search strategy

Table S2. Study eligibility criteria

Table S1. Literature search strategy

Database	Search	No. of articles
PubMed (MEDLINE)	(“magnesium depletion score” [title/abstract]) AND (english[Filter]) NOT (animals [mh] NOT (humans [mh] AND animals[mh]))	48
Embase	'magnesium depletion score':ti,ab AND 'english':la NOT ([animals]/lim NOT [humans]/lim)	44
Scopus	'magnesium depletion score':ti,ab AND 'english':la NOT ([animals]/lim NOT [humans]/lim)	47
CINAHL	TI "magnesium depletion score" OR AB "magnesium depletion score"	7
Web of Science	TI "magnesium depletion score" OR AB "magnesium depletion score"	60

Table S2. Study eligibility criteria

Category	Inclusion criteria	Exclusion criteria
Study design	Clinical trials	*In vitro* studies
	Observational studies	Animal studies
		Reviews, letters to the editor, editorials, commentaries, conference abstracts, and conference proceedings
		Scoping reviews
		Systematic reviews and/or meta-analyses
		Umbrella reviews
Study duration	No restriction	No restriction
	No restriction	No restriction
Age of participants	No restriction	No restriction
Population health status	No restriction	No restriction
Intervention/exposure	Magnesium depletion score	No restriction
Outcomes	No restriction	No restriction
Comparator	No restriction	No restriction
Date of publication	No restriction	No restriction
Publication status	Published peer-reviewed articles	Non-peer-reviewed articles, unpublished data, preprints, and retracted articles
Language	Published in English	Languages other than English
Country	No restriction	No restriction
